# Nutrition modeling tools: a qualitative study of influence on policy decision making and determining factors

**DOI:** 10.1111/nyas.14778

**Published:** 2022-04-20

**Authors:** Frances Knight, Megan W. Bourassa, Elaine Ferguson, Helen Walls, Saskia de Pee, Stephen Vosti, Homero Martinez, Carol Levin, Monica Woldt, Kavita Sethurman, Gilles Bergeron

**Affiliations:** ^1^ Department of Population Health London School of Hygiene and Tropical Medicine London United Kingdom; ^2^ Nutrition Division United Nations World Food Programme Rome Italy; ^3^ New York Academy of Sciences New York New York; ^4^ Friedman School of Nutrition Science and Policy Tufts University Boston Massachusetts; ^5^ Human Nutrition Wageningen University Wageningen the Netherlands; ^6^ Department of Agricultural and Resource Economics University of California, Davis Davis California; ^7^ Nutrition International Ottawa Ontario Canada; ^8^ Department of Global Health University of Washington Seattle Washington; ^9^ Helen Keller International Washington District of Columbia; ^10^ USAID Advancing Nutrition Arlington Virginia; ^11^ Formerly with Food and Nutrition Technical Assistance Project (FANTA) Washington District of Columbia; ^12^ Poverty, Health, and Nutrition Division International Food Policy Research Institute Washington District of Columbia

**Keywords:** nutrition modeling tools, policy‐making processes, decision makers, linear programming

## Abstract

Nutrition modeling tools (NMTs) generate evidence to inform policy and program decision making; however, the literature is generally limited to modeling methods and results, rather than use cases and their impacts. We aimed to document the policy influences of 12 NMTs and identify factors influencing them. We conducted semistructured interviews with 109 informants from 30 low‐ and middle‐income country case studies and used thematic analysis to understand the data. NMTs were mostly applied by international organizations to inform national government decision making. NMT applications contributed to enabling environments for nutrition and influenced program design and policy in most cases; however, this influence could be strengthened. Influence was shaped by processes for applying the NMTs; ownership of the analysis and data inputs, and capacity building in NMT methods, encouraged uptake. Targeting evidence generation at specific policy cycle stages promoted uptake; however, where advocacy capacity allowed, modeling was embedded ad hoc into emerging policy discussions and had broader influence. Meanwhile, external factors, such as political change and resource constraints of local partner organizations, challenged NMT implementation. Importantly, policy uptake was never the result of NMTs exclusively, indicating they should be nested persistently and strategically within the wider evidence and advocacy continuum, rather than being stand‐alone activities.

## Introduction

Appreciation of the role of nutrition in human, social, and economic development has led to increased nutrition‐focused policy, public investment, and donor contributions in low‐ and middle‐income countries (LMICs).^1^, ^2^ Current resourcing remains insufficient, however, and advocacy to strengthen commitments long‐term is needed, as is the concurrent prioritization of the most urgent and most effective nutrition issues and programs to receive limited resources.^2^ There is a recognized need for the rapid generation of context‐specific evidence to answer relevant questions for nutrition policy and program decision making and inform resource prioritization, guide regulatory and evaluation activities, and encourage further commitment and investment toward improving nutrition.^3^, ^4^, ^5^

A number of nutrition modeling tools (NMTs) have been developed by researchers working in academic institutions and international organizations to generate the evidence needed to answer some of the questions relevant at different nutrition policy and program cycle stages. Most of these NMTs differ in terms of data inputs, analytical methods, and results that can be generated, but are common in that they apply mathematical modeling and are intended to be adopted and used, or accepted and supported, by decision makers or their advisors to inform nutrition policy and programs in LMICs. Several NMT developers are members of the Nutrition Modeling Consortium (NMC), which aims to increase the connection between NMTs and to encourage the uptake of results.^2^ An overview of the Consortium and functions that can be performed by the different tools is provided on the NMC website (https://www.nyas.org/programs/nutrition‐modeling‐consortium/).

Despite often widespread application of NMTs, the literature to date has been largely limited to model development, methods, and modeling results from individual analyses, rather than how the tools and evidence they generate are applied and any influence this has had on policy or program decisions.^6^, ^7^, ^8^, ^9^, ^10^ Anecdotally, NMC members have shared that NMT results have not always had the desired use or program or policy influence in‐country, and that there is a need to understand how to strengthen this. Simultaneously, there is increasing pressure on researchers and technical assistance providers to demonstrate the impact of their evidence‐generation methods on policy processes and outcomes to justify approaches and resource allocation and encourage decision makers in other contexts to apply similar methods.^11^ However, attributing policy change to a single piece of evidence is difficult and mostly unrealistic.^12^, ^13^

Individual analyses are unlikely to be comprehensive or convincing enough to be used as the only rationale for significant policy and related investment decisions since additional questions will inevitably remain.^11^, ^12^, ^14^, ^15^ Therefore, the influence of individual pieces of evidence, or the ability to measure their impact in a simple way, should not be exaggerated.^11^ That said, evidence can be catalytic in kicking off new discussions or nudging forward slow or stalled policy processes, which could contribute to significant policy decisions.^12^, ^13^ NMT applications may not have directly resulted in changes to policy implementation, yet the evidence generated could have been instrumental in changing the framework in which nutrition problems and solutions are viewed and shaping aspects of the policy process, including generating consensus, creating an enabling environment for a cause, or contesting proposed actions or positions.^13^, ^14^, ^16^, ^17^

For the above reasons, rather than attempt to objectively measure and compare policy impact across NMTs or NMT applications, the aim of this study was to draw on user experiences from a large selection of NMT case studies to document reported decision‐making applications of the tools and their results and identify internal and external factors to the tool application process that limit or enhance the extent to which the modeling is considered and used by decision makers. Furthermore, this study explores end‐user perceptions of NMTs and their use in general and does not seek to measure and compare the potential and accomplishments of individual tools. NMTs relevant to this study and the program and policy questions they can answer are outlined in Table 1 and Table S1 (online only).^7^, ^10^, ^18^, ^19^, ^20^, ^21^, ^22^

**Table 1:** Policy and program cycle stages, key questions, and nutrition modeling tools capable of answering these questions examined in this study

		Nutrition modeling tool											
Policy or program stage	Key technical questions for policymakers and program designers	CoDB	CoHA	CoNBF	PROFILES	MAPS	Optifood	CotD	FNG	LiST	MINIMOD	IMAPP	SEEMS
1. Advocacy	What are the social and economic costs of malnutrition?	X	X	X	X								
2. Situation assessment	How does nutrient supply and status vary spatially?					X					X		
	Can nutrient requirements be met by local food systems?						X						
	How accessible are nutritious diets?							X	X				
3. Prioritization	Which nutrition issues should be prioritized?				X					X			
	Which groups and geographies should be targeted?					X			X		X		
4. Policy or program review and development	How much could nutrient adequacy be improved using specific foods or nutrients?					X	X		X		X	X	
	Which combination of interventions could have the greatest impact on health outcomes?									X	X		
	Which multisectoral actions could most improve access to nutritious diets?								X				
5. Resource allocation and planning	How much would it cost to improve selected nutrition indicators in terms of program costs?					X					X		
	Which interventions would be most cost‐effective?										X		X
6. Evaluation	What impact have program likely had on nutrition outcomes?						X	X	X			X	X

*Note*: See Table S1 (online only) for a complete list and descriptions of the nutrition modeling tools examined.

CoDB, Cost of the Double Burden; CoHA, Cost of Hunger; CoNBF, Cost of Not Breastfeeding; CotD, Cost of the Diet; FNG, Fill the Nutrient Gap; IMAPP, Intake Modeling, Assessment and Prediction Program; LiST, Lives Saved Tool – Nutrition; MAPS, Micronutrient Action Policy Support; SEEMS, Strengthening Economic Evaluation of Multisector Strategies for Nutrition.

## Methods

This qualitative study involved semistructured interviews with end users of 12 different NMTs across 22 LMICs (Table 2). Nutrition Modeling Consortium members listed all applications of their tools in which results were disseminated in the country of application within the past 7 years. This time period was set to encompass applications of all relevant NMTs while still being recent enough for respondents to recall details of the activity. A target of three country case studies per NMT was set to capture different analysis processes and geographical variation. Fewer case studies were targeted for tools that were new or still under development, and two of the 14 NMC tools were excluded since appropriate case studies could not be identified. Thirty case studies were then randomly selected from the list of NMT applications. The tools represented by the case studies were: Cost of the Double Burden;^23^ Cost of Hunger;^24^ Cost of Not Breastfeeding;^22^ Cost of the Diet;^25^ Fill the Nutrient Gap;^26^ Intake Modeling, Assessment and Prediction Program (IMAPP);^27^ Lives Saved Tool – Nutrition (LiST‐Nutrition);^28^, ^29^ Micronutrient Action Policy Support (MAPS);^30^ MINIMOD;^21^, ^31^, ^32^ Optifood;^18^, ^19^ PROFILES Nutrition Advocacy Tool;^33^ and Strengthening Economic Evaluation of Multisector Strategies for Nutrition (SEEMS‐Nutrition).^34^

**Table 2:** Number of case studies per nutrition modeling tool and by geographical region

	Geographical region							
Nutrition modeling tool (NMT)	Central Asia	East Africa	Latin America	South Asia	South East Asia	Southern Africa	West Africa	Total
CoDB			3					3
CoHA		1				2		3
CoNBF					1		2	3
CotD*^a^*		1		1	1			3
FNG*^b^*	1				1		1	3
IMAPP		1						1
LiST Nutrition						2	1	3
MAPS						1		1
MINIMOD		1	1			1	4	3
Optifood		1	1	1				3
PROFILES		1				2		3
SEEMS				1				1
Total	1	6	4	3	3	7	6	30

CoDB, Cost of the Double Burden; CoHA, Cost of Hunger; CoNBF, Cost of Not Breastfeeding; CotD, Cost of the Diet; FNG, Fill the Nutrient Gap; IMAPP, Intake Modeling, Assessment and Prediction Program; LiST Nutrition, Lives Saved Tool – Nutrition; MAPS, Micronutrient Action Policy Support; SEEMS, Strengthening Economic Evaluation of Multisector Strategies for Nutrition.

^*a*^ The CotD case studies included in this study refer to CotD applications only, using the Save the Children UK methods and were not related to FNG applications.

^*b*^ The FNG applications included diet modeling in the CotD tool, according to FNG methodology.^26^

Interview participants were selected using purposive and snowball sampling^35^ to represent three end‐user groups for each case study, as defined by NMC members:^36^ (1) “Brokers” who know about an NMT and may recommend, coordinate, or fund its application; (2) “Technical analysts” who conduct analysis using the tools; and (3) “Consumers” who would ideally use the modeling to inform advocacy and decision making (Table 3). Sampling continued until all end‐user categories were represented and information saturation occurred, in terms of new data repeating what had been expressed in previous interviews.^35^, ^37^ In total, 109 key informant interviews were conducted with: technical‐ and policy‐level government staff (*n* = 25) from national health, planning, and agriculture ministries; local and international program staff from United Nations (UN) agencies (*n* = 34); nongovernmental organizations (NGOs) (*n* = 31); and academic researchers (*n* = 19) (Table 4). Although there were no recorded refusals, participation was affected by connectivity and scheduling challenges.

**Table 3:** End‐user categories and definitions

End user	Definition used	Examples
Brokers	Individuals or organizations who know about a tool and connect people (analysts and consumers) with them and may recommend, coordinate, or fund the tool application.	Country‐based representatives from donor organizations, and managers and nutrition staff from international organizations managing a tool application process.
Technical analysts	Those who conduct an analysis using a nutrition modeling tool.	Local technical nutritionists, epidemiologists, economists, or other analysts and international specialists, researchers, or consultants.
Consumers	Policymakers and program planners (or their advisors) who may ask initial analysis questions and would ideally use the modeling results to inform decision making or for advocacy.	Management and higher‐level government staff, including SUN coordinators, government unit directors, and ministers, as well as locally based managers or directors from NGOs or other organizations.

**Table 4:** Number of interviews by end‐user group, case‐study location, affiliation, and sex

	Broker	Technical analyst	Consumer	Total
Case‐study location				
Central Asia	2	2	2	6
East Africa	5	7	5	17
Latin America	8	4	4	16
South Asia	2	4	1	7
Southeast Asia	6	4	8	18
Southern Africa	9	8	9	26
West Africa	7	7	5	19
Affiliation				
Academia	4	12	3	19
Government	2	6	17	25
NGO	13	10	8	31
UN agency	20	8	6	34
Sex				
Female	26	19	20	65
Male	13	17	14	44
Total	39	36	34	109

The conceptual framework for the study was derived from a review of gray literature describing NMT applications and policy influence, NMC meeting reports, and scoping interviews with NMC members. Six key concepts (reasons for analysis, analysis process, stakeholder involvement, dissemination, use of results for advocacy or decision making, and factors influencing this) were used to develop the interview guide (Table S2, online only) and later to structure the analysis. Interviews included several open‐ended questions on each of the following topics:

- Reasons for selecting the tool that was used and analysis objectives (Q2 and Q3)
- Processes for engaging and working with stakeholders (Q4 and Q5)
- Process for planning and carrying out the analysis (Q6–Q9)
- Recall of and reaction to modeling results and recommendations (Q10)
- Processes for disseminating and applying results (Q11–Q13)
- Perceived influence of the modeling and factors that influenced this (Q14–Q24)

Interviews were conducted by the first author between August 2018 and March 2020, mostly in English, otherwise in Spanish or Portuguese, in person or via phone/web conference. Participants were provided with an information sheet and asked to provide consent after discussing study aims, confidentiality provisions, and any questions. Interviews were digitally recorded with permission, transcribed verbatim and, if necessary, translated into English. Detailed notes were taken when recordings were not possible. Transcripts and notes were checked and edited to aid understanding. NVivo 12 was used to organize, code, and analyze deidentified data from the interviews.^38^ Approval for this study was obtained from the LSHTM Research Ethics Committee (ref. 16729).

The data were explored using reflexive thematic analysis (RTA), which is useful for the categorization of large datasets and to examine the experiences, knowledge, and opinions of diverse stakeholders.^39^, ^40^ In RTA, themes are created inductively and deductively throughout the analytical work as opposed to being found in the data alone, meaning the approach is flexible and allows the development, review, and sorting of codes and data across different stages of the analysis.^39^, ^40^ The analysis followed the six‐step RTA process outlined by Braun and Clarke, with movement back and forth between steps.^39^, ^40^, ^41^, ^42^ (1) Interview transcripts and notes were reviewed to provide familiarity with the data. (2) Data were deductively coded using broad codes from the conceptual framework;^41^, ^42^ more detailed codes were then developed inductively and applied semantically to explore topics raised in the interviews.^41^, ^42^ (3) Themes, defined as patterns of shared meaning underpinned by a central concept or idea, were generated by grouping codes and coded data.^39^, ^40^ (4) Transcripts and notes were reread, and themes edited to ensure coding completeness and that the themes were useful and accurately represented data. (5) The scope and focus of each theme was defined and named. (6) Demonstrative quotes were selected and organized by theme to highlight findings across end‐user groups.

Tools and case‐study countries were not identified in the analysis in order to protect the identity of participants and avoid comparison of the features and methods of tools themselves or organizations applying them. Instead, case studies were grouped by their geographical regions, policy stage, and general tool characteristics (e.g., tools that translate nutrition issues to social cost).

Results are presented across the three following sections: (1) tool application processes, including reasons for selecting NMTs, processes for engaging and working with stakeholders, planning, carrying out and disseminating analysis; (2) analysis objectives and influence, covering analysis objectives, reaction to and use of results, and perceived influence of these; and (3) internal and external factors affecting the use of modeling results and their influence on program and policy decision making.

## Results

### Processes for applying NMTs

The case studies were varied in terms of reasons for selecting the NMTs that were used, partners involved, who did the analysis, and how results were disseminated. While the countries using the tools were LMICs, most analyses were initiated by international NGOs, UN agencies, and European or North American universities. Occasionally, NMTs were applied in response to government requests to these organizations for assistance to answer key policy questions or use data that had been collected for another purpose. This generally happened when government partners were already aware of analyses that had previously been done by these organizations or their particular technical capacity. In very few cases, there was existing capacity to use a particular tool in country or knowledge about one or more NMTs, which prompted tool use. Government inputs at this stage were predominantly focused on whether to endorse a proposed NMT analysis, instead of deciding which tool could answer necessary policy questions.

Government partners oversaw, conducted, or validated NMT analyses in nearly all cases, especially when they aimed to influence national decision making. Table 5 summarizes six distinct processes for technical government partners or other local entities to engage with NMTs or their NGO, UN, or academic brokers. These included: (1) via preloaded online dashboards; (2) independently accessing and using NMTs; (3) being trained and supported to apply NMTs on an ongoing basis; (4) being trained to use an NMT for a specific analysis; (5) preparing inputs for analyses done by international specialists; and (6) reviewing analyses conducted by international specialists. With the exception of NMTs with preloaded dashboards, the engagement process was mostly determined by context and stakeholder preferences, with some tools applied differently across case studies.

**Table 5:** Summary of approaches for engagement and analysis with NMTs reported in the case studies

Process for engaging with NMT	Findings	Tool types	Intended local users of NMTs or modeling outputs (MOs)
1. Analysis presented on preloaded, online dashboards.	Used by few NMTs that process inputs from secondary datasets (DHS, etc.). Considered interactive alternative to static reports	Those answering single policy questions applied once/every 4–8 years. Limited user input required for standard analyses, yet custom data entry possible for some.	Policymakers or those advising policymakers (nontechnical) (NMT).
2. Analysts access tool and conduct modeling independently.	Very infrequent. Some analysts reported difficulty in accessing technical support to troubleshoot issues.	Well‐established tools with online training and support materials.	Technical analysts with nutrition knowledge (NMT).
3. Analysts trained and supported to apply NMT as part of ongoing monitoring or research activities.	Training and support provided in some cases for existing or purpose‐formed groups to apply NMTs, for monitoring or to support policymakers as new data became available or policy questions emerged.	Tools applicable at different points in time/to answer emerging policy questions. Established tools with the resources to provide intensive training and support.	Technical analysts with nutrition knowledge (NMT).
4. Analysts trained at one point in time to conduct modeling, independently or with support.	Common approach. Many government and nongovernmental analysts trained. In some cases, trainers oversaw and supported analysis also. Occasionally tools institutionalized into local university programs. Capacity difficult to sustain due to staff turnover and infrequent use.	Well‐established tools with manuals or tools for which intensive training could be provided, locally or internationally (resources needed).	Technical analysts with nutrition knowledge (NMT).
5. Local partners prepare modeling inputs for international specialists to run analysis.	Relevant to some tools. Local analysts prepare or validate inputs and review results. Any training provided focused on understanding methods and data requirements.	Tools with relatively straightforward data inputs, for example, Excel‐based tools answering single policy questions.	Technical nutrition staff and stakeholders representing non‐nutrition sectors (MOs).
6. International researchers or consultants run analysis with local review.	Common approach for modeling done by international NGOs, UN organizations, or universities. Overviews provided to help stakeholders understand methods and findings. Technical partners often provided inputs and reviewed results via working groups.	Complex tools asking multiple policy questions, where standard approaches are required to allow comparison across settings or new tools for which supporting material not yet available.	Technical and nontechnical staff representing nutrition and non‐nutrition sectors (MOs).

*A consultant came from (International University) to train a group of local researchers and analysts. We invited partners we thought may be interested*.

NGO Broker 089, South Asia

*The Technical Working Group was people from academia, WHO, UNICEF, Ministry of Trade and Industry, Agriculture, and Health. We discussed all of the numbers that went into the analysis. We had to have consensus*.

NGO Technical Analyst 107, East Africa

NGO or UN brokers commonly described the NMT processes as being government “owned,” a term which would generally imply that local stakeholders regard an analysis as theirs and are closely involved in its planning, implementation, and dissemination.^43^ Explanations of how ownership was achieved, however, ranged from nominating a local analysis champion, such as a Scaling Up Nutrition (SUN) Focal Point, to appointing a government‐led technical working group to review and validate analysis inputs and outputs, to government partners doing the analysis themselves. Nutrition policy mapping exercises were done in a few cases to identify relevant stakeholders and decision makers to involve in the analysis or dissemination and determine how and when they should participate.

*We (SUN Coordinator and team) were involved from the beginning, engaging partners and facilitating the whole process. We worked closely with the analysis team*.

Government Consumer 018, Southern Africa

Local analysts were trained and supported to conduct the analysis in several cases, where practical, which was generally considered preferable to the more common approach of international specialists doing analyses. Issues of sustainability, both in terms of reliance on outsiders and maintaining local capacity, were raised by many respondents. In some contexts, “divisions of labor” were identified to facilitate the analysis and also promote understanding of the methods and involvement of local partners.

In some cases, the NMT process expanded beyond analysis and dissemination to actively translating evidence from the modeling into advocacy. Communications and advocacy specialists occasionally supported such activities, but elsewhere, expertise in these areas and resources were lacking. Some analysts were frustrated that advocacy based on NMT results and the development of materials to support this could take significantly more time and resources than analysis, yet were not prioritized or planned for.

*The main product was a manuscript, but we also did a lot of work converting it (modeling) into very quick, simple presentations for high‐level decision makers*.

Government Consumer 024, Southern Africa

*A meeting was organized through high‐level officials, and the results were used to make the case and really remind the parliamentarians of what they had committed to*.

NGO Broker 028, West Africa

### Analysis objectives and influence

Objectives and reported uses or influences of the modeling are summarized in Table 6, by policy or program cycle stage. Almost all NMT analyses were done to inform government decision making and some simultaneously targeted multiple entry points, for example, encouraging commitments to addressing malnutrition long‐term while informing the specific program designs short‐term.

**Table 6:** Analysis objectives, use, reported policy and program influences, and findings from examined case studies, arranged by intended policy and program cycle stages

Cycle stage	Analysis objectives	Use	NMT policy and program influences	Findings
1. Advocacy	Encourage awareness and commitment	Broad, government‐focused objectives common, especially for NMTs estimating economic and social costs of malnutrition.	Increased commitment	Many consumers saw analysis as a catalyst (among others) for new commitments, for example, inclusion of nutrition in national development plan or a nutrition task force.
			Validated existing policy or program	Rarely, analysis affirmed existing policy or actions. Considered strategic for maintaining high‐level nutrition commitments and funding.
			Informed general advocacy	Often used by government and occasionally other partners to advocate for attention to and investment in nutrition in general.
	Provide evidence for targeted advocacy	In a few cases, NMTs used to inform or strengthen ongoing advocacy campaigns.	Contributed to specific advocacy	In the few cases where this was an objective, nongovernmental actors translated modeling into messages targeting government, for example, demonstrating economic benefits of breastfeeding within larger breastfeeding promotion campaign.
	Positioning of organization	Occasional secondary objective for nongovernmental agencies.	Gained a seat at the table	In a few cases, analysis considered important for reputation and relationships with government. Credited with invitations to government policy discussions.
2. Situation assessment	Characterize nutrition situation	In some cases, NMTs used to provide updates on the nutrition situation and understand causal factors.	Enhanced understanding	Credited for addressing misconceptions and improving understanding of determinants by nutrition and non‐nutrition stakeholders, for example, debunking beliefs that child malnutrition is solely due to poor care practices and showing nutritional impacts of food price shocks.
3. Prioritization	Prioritize specific nutrition issues	Many analyses done to identify and draw attention to issues, groups, or areas of greatest opportunity for impact for government or nongovernmental policy and programs.	Put specific issues on agenda	Often, individual issues, such as nutritious diet access or rice fortification, said to gain momentum and be spoken about more by stakeholders following NMT analysis.
			Prioritization of vulnerable groups	Occasionally, vulnerabilities highlighted by the modeling were targeted in subsequent policy with tailored interventions or prioritized in social protection targeting or supplementation programs.
4. Policy or program review, development, and planning	Inform government prioritization of nutrition programs	Analyses often modeled and compared cost and/or potential impact of alternative nutrition‐specific or ‐sensitive interventions to inform their prioritization in government policy	Informed government planning	Often informed selection or design of specific actions, mostly in national sector‐specific and multisectoral policy, for example, which interventions, where, and for whom? Results often referenced in policy as justification, for example, for fortification legislation.
			Used to advocate for specific programming changes	In a few cases, government nutrition stakeholders used modeling in extra‐ministerial advocacy for commitments or funding. Elsewhere, used by NGOs/UN to appeal for specific changes to government programs (e.g., use of fortified rice or cash transfer amounts).
	Inform prioritization of nongovernmental actions	NMTs occasionally used to inform NGO or prioritization of activities, generally secondary to objectives to informing government actions.	Informed design and justification of programs	For some cases with government policy as the primary objective, modeling also used to design, make a case internally, or obtain funding for nongovernmental programs by demonstrating need and/or potential effectiveness. Many analyses linked to a greater inclusion of nutrition‐sensitive actions in NGO/UN country strategies or work plans and used in funding applications.
			Applied to improve existing programs	Occasionally, nongovernmental organizations changed existing programs as a result of modeling findings, for example, composition of fortified products or foods promoted for homestead production.
	Inform design of specific programme components	NMTs applied in several cases to compare and select between programme components.	Informed key design decisions for new programmes	Almost all analyses done to inform NGO/UN programmes used to design specific programme elements (SBCC messages, portion sizes of complementary foods, transfer values, fortification vehicles, etc.). In a few cases, informed government programme elements and rarely, contributed, along with other evidence, to justifying scale‐up successful interventions.
5. Resource allocation	Estimate programme cost–benefit	Very occasionally modeled benefits linked with implementation costs to demonstrate programme value and encourage uptake.	Influenced programme scale‐up	For the few tool applications, applying cost–benefit analysis, modeling instrumental in decisions reprogramme scale‐up, for example, government adoption and extension of an existing NGO programme.
6. Evaluation	Assess extent to which situation may have changed as a result of programme	Occasionally analyses used data from different time periods to indirectly estimate extent to which situation had changed, or needs had evolved.	Informed updates to programme elements	Primarily used to justify alterations to existing programmes to meet objectives. Most concerned justifying increases to cash transfer amounts in livelihood or social protection programmes.
			Used to appeal for resource adjustments	In some cases, modeling justified government or donor approval for updates to programme elements to enhance impact on nutrition or better respond to identified needs, such as funding allocation to increase programme coverage or dose.

Tools translating malnutrition into social, economic, and human capital costs were almost always applied to encourage awareness and investment in nutrition and often timed to coincide with political transitions or national budget planning.

*We have a new government administration. We wanted to use the modeling to show that nutrition should be top of their list for development priorities*.

UN Broker 009, Latin America

Many consumers reported that the modeling, alongside other evidence and advocacy, influenced government prioritization of nutrition, for example, commitments to nutrition in national development plans, malnutrition task forces, or the introduction of nutrition teams in nonhealth ministries.

*The approval for the national food security and nutrition council was based on this (modeling)*.

Government Consumer 023, Southern Africa

Occasionally, consumers said modeling substantiated existing policy, which helped ensure continued funding. Elsewhere, the modeling was being applied as part of ongoing advocacy efforts for specific goals.

*Even if the analysis only served to validate existing policy, this was valuable because the policies prioritizing nutrition from the Ministry of Health, Agriculture, etc. exist, but strategies are not always funded. We used the results to make a case to Treasury*.

Government Consumer 014, East Africa

The analysis was important because even though the country has a strong policy focus on malnutrition, with this economic situation we‘re promising more than we can actually afford. So, they (Treasury) are looking for things to cut and we need to prove why we need to keep them.

Government Consumer 007, Latin America

A few analyses were important for engagement and credited with intergovernmental agencies and NGOs gaining a seat at the table for discussions at subsequent stages of a policy cycle.

*The (analysis) enabled (organization) to get a seat at the table on policymaking. This helped us insert recommendations supporting the Multisectoral Nutrition Action Plan*.

UN Broker 073, Southeast Asia

A few analyses were done for situation assessment, to complement health and nutrition surveys, and to better understand factors contributing to, or the extent of, the malnutrition burden. Some analyses were credited with addressing misconceptions, which led to a more favorable environment for appropriate policy responses.

*In the common mind of government, people had malnourished children because they were “misbehaving” and not feeding them properly, but now we have facts and can show that there are issues with access to nutritious diets*.

UN Broker 045, West Africa

*The Minister of Finance was shocked! I remember him saying “how come I didn't know that this was the impact (of malnutrition) on the economy, and that I could save so much money just by making sure kids eat okay.”*

UN Broker 017, Southern Africa

A number of analyses were done to prioritize the specific needs of particular groups or geographies. This was common in contexts with significant subnational diversity and limited resources and was often a precursor to further modeling to compare the potential effectiveness of different program options. Issues or groups highlighted by the modeling commonly gained momentum and were spoken about more by stakeholders. In a few examples, new policies or programs were introduced by government or others to target vulnerabilities focused on by the analysis.

*The high prevalence of deficiencies we showed was confirmed by the UNICEF SMART survey. The modeling convinced the government to define the northern region as the most vulnerable. So now when there is not enough money for Vitamin A campaigns everywhere, they will make sure at least this region gets them*.

NGO Broker 081, West Africa

*Now we see more intensive nutrition programs for adolescent girls. This may have happened anyway, but the additional evidence helps. Because here people say “where's the evidence?” so it strengthens the argument for who you look after when we talk about nutrition*.

Government Consumer 054, Southeast Asia

Analyses at the policy or program review and development stage mostly sought to guide government decisions on which nutrition‐sensitive or ‐specific actions to implement, where, and targeted toward whom. Few analyses focused on nongovernmental programming alone, although some included models to inform decisions for both government and nongovernmental bodies. Modeling compared the potential impacts of actual or proposed health, agriculture, food security, or social protection policies or programs on outcomes, such as nutrient intake, lives saved, nutritious diet access, and prevalence of inadequacy. The modeling was occasionally mentioned as justification for decisions, including mandatory fortification legislation, regulation on processed food use in school feeding, or inclusion of nutritious foods in social protection programs.

*We did analyses on under‐five mortality, stunting, and wasting reduction. We proposed five intervention scenarios, some that were being used and others that were new. The Minister of Health used our models to develop projections for a 10‐year action plan*.

Academic Technical Analyst 071, West Africa

*The government approved the ruling on imported oil; now the oil coming to this country will be fortified with Vitamin A*.

Government Technical Analyst 067, East Africa

Elsewhere, technical‐level government advisors used the modeling to advocate for specific changes, such as fortification standards or social protection transfer amounts. In many instances, however, local nongovernmental partners used the modeling for advocacy.

*She (technical government colleague) asked for support to transition from periodic Vitamin A campaigns to routine distribution. This request has to come from the minister. So, we are working on a presentation to help her convince the minister first*.

Academic Broker 082, West Africa

*The modeling gave us authority to talk to government and the big social protection partners like UNICEF and the World Bank. We were able to get the government to push up the cash transfer amount. We could never reach what was actually needed to meet the nutrient requirements but at least we increased it*.

NGO Consumer 042, Southeast Asia

Within NGOs, modeling was used to convince non‐nutrition colleagues and donors of the value of nutrition‐sensitive programming, to sustain existing programs, and introduce new components. Examples included local procurement of nutritious foods for school feeding, nutrition behavior change messages linked to cash transfers, and extending food voucher eligibility to include nutritionally vulnerable adolescent girls.

*It (modeling) showed the economic benefits of providing more nutritious school meals and linking with activities that could provide income for the local community. Now we're supporting small livestock and vegetable production and part of the food is used in schools*.

UN Consumer 044, West Africa

*Supplements in the school feeding, that was already done before the analysis, so the modeling didn't change what was being done but it did mean that we kept doing it. We could show the logistics and procurement teams it was worth the effort and demonstrate value to the donors and stakeholders*.

UN Consumer 051, Southeast Asia

A few NMT analyses estimated cost–benefit using data from pilot or early phase program implementation to link observed benefits with implementation costs. Such modeling informed program adoption and expansion, such as the government scale‐up of an NGO pilot incorporating agricultural, early childhood development, and school feeding actions.

*The data that we are generating is going into the government planning for the scale‐up. When they roll out, they'll have more data coming back in to continuously inform the program*.

Academic Technical Analyst 109, Southern Africa

Occasionally, NMTs contributed to program evaluations, predicting the extent to which selected indicators may have changed by retroactively comparing baseline models with those based on new data. This demonstrated likely program value and identified or justified necessary alterations. In some cases, design changes required approval or further resources and the modeling was considered important for making a case to decision makers.

*There was a budget excess and the donors wanted to reach more people. We're using the modeling to make a case for improving program quality instead. We agreed to give both animal‐source foods and vegetables; previously households got only one or the other, so now we are doubling the support in a way*.

NGO Technical Analyst 037, South Asia

### Factors influencing the use of NMT results

Table 7 summarizes 10 factors most commonly reported as influencing the extent to which modeling was accepted and used by decision makers. We distinguish between internal factors that were largely within the control of tool users and external factors.

**Table 7:** Summary of factors found to influence the use of NMT modeling results to inform policy decision making

	Factor	Findings	Considerations
Internal	1. Reputation and acceptance of NMTs	Origin, scientific validity, and perceived acceptance of NMTs by the international community important to all policymakers interviewed. Examples of tool applications and evidence of academic merit influenced decisions to use NMTs or government approval for analyses.	Demonstrate validity of tools and methods and share examples from other settings, especially regional. Publish tool methods and applications widely.
	2. Targeting analysis at key decisions and policymakers	End users credited strategic targeting of analysis objectives and dissemination at key policy areas and policymakers with uptake and impact of the modeling. This often meant engaging with nontraditional nutrition stakeholders.	Define intended policy applications and target analysis processes, dissemination materials, and advocacy activities accordingly.
	3. Timing of analysis	Alignment with particular policy or program cycle stages, such as development of a new national nutrition plan, considered critical by many. Less relevant for NMTs with multisectoral applications as cycles differ across ministries. Instead, strategic advocacy needed to insert modeling into emerging policy discussions opportunistically.	Ensure timing of analysis in line with targeted policy processes, if relevant, but continue to see opportunities to use modeling in policy discussions.
	4. Perceived complexity of NMTs	Many considered NMT methods complex and difficult to explain. Consumer end users sometimes misunderstood modeling outputs or felt unconfident presenting them to decision makers or applying them to policy.	Present NMT methods and results at multiple occasions in different formats. Involve partners in the analysis and presentations.
	5. Ownership of analysis process	Ownership critical for uptake of modeling. Meaningful involvement of stakeholders in decisions about analysis inputs and outputs, if not the analysis itself, seen as minimum standard by many consumers for analyses targeting government policy.	Engage with local stakeholders to reach a shared understanding of ownership and how to ensure it. Include local partners in analysis process.
	6. Building local capacity to apply NMTs	All respondents felt positively about local actors carrying out or supporting NMT analyses, yet views on training fell evenly into three categories: (1) local analysts should always be trained and apply NMTs independently, (2) capacity is unlikely to be sustainable enough to justify the investment to train local analysts, and (3) training could be appropriate in some cases but advocacy could be a more pressing capacity need.	Capacity building for NMTs important; depends on context, stakeholder preferences, tool characteristics, and other capacity‐building needs. Embedding NMTs in local academic or government institutions could encourage sustainability.
	7. Use of secondary data	Modeling inputs derived from government or other secondary data viewed positively by many consumers as they were considered reputable, promoted ownership, and validated government surveys. Also, this often meant the analysis could be done relatively quickly and inexpensively, compared to collecting and then analyzing primary data.	Use secondary data as agreed with local stakeholders to develop or validate NMT analysis inputs where feasible and practical.
External	8. Capacity for evidence translation and advocacy	Ability to translate modeling evidence into advocacy action and apply evidence to decision making considered critical for policy impact. Low capacity in this often reason for poor use or follow up to the analysis. Include capacity to develop and promote advocacy messages and identify opportunities for strategic application of the modeling.	Plan and budget for development of advocacy strategies and messages based on NMT analysis. Assess and support capacity of partners responsible for acting on the NMT results.
	9. Resources for local institutions to support analysis	Partners were often overburdened and lacked time, human resources, and funds to adequately participate in the analysis process and apply modeling to advocacy and policy. Funding for tool applications rarely provided to government partners themselves.	Communicate and agree upon realistic responsibilities prior to commencing analysis. Consider appointing staff (and resources) at local partner institutions to support NMT processes.
	10. Political change	Challenge for most case studies and more broadly in government and development. Affected participation in the analysis process and commitments or momentum for the advocacy efforts it contributed to.	Involve multiple partners in analysis and dissemination to avoid dependence on individuals.

#### ***Internal factors***. Reputation and acceptance of methods

Reputation of tools and their methods was instrumental; some brokers and consumers supported proposals to use a tool because the methods had been published in peer‐reviewed journals and or was considered to have academic acceptance. Examples of a tool's successful use in neighboring countries also encouraged acceptance and uptake. Existing relationships were influential and government policymakers valued the opinion of donor and UN counterparts. Therefore, if such counterparts had previous knowledge or an opinion about a particular tool, positive or negative, this could influence government decisions to approve an analysis process or accept NMT findings.

As such, it was important that brokers consulted widely to obtain support for NMT analyses.

*We used the results from Mexico, Ecuador, and Chile to get support for the (NMT) study. This was important as the examples were from our region*.

Government Consumer 001, Latin America

#### Targeting

Conscious messaging and targeting of key stakeholders during the analysis process and delivery of outputs was commonly mentioned as a success factor. Brokers strategically identified technical partners, analysis “owners,” and advocacy recipients based on intended use and influence of the modeling. As such, some analysis working groups were led by agriculture or education departments rather than traditional nutrition partners, such as health. Similarly, at dissemination, finance ministers, treasury, and regional government leaders, who could influence resource allocation, were often singled out with specific materials. Occasionally, this was recognized by government consumers who praised the presentation of nutrition issues in a language that was relevant to them, such as economic cost.

*Partners need to match the focus of the modeling but also have the capacity to move things. The “leading organization” was not Health but the Ministry of Education for school feeding*.

UN Broker 046, West Africa

*You need to convince those in charge of budgets, who aren't natural nutrition stakeholders. You need incredibly clear, simple, and persuasive evidence and materials*.

Government Consumer 088, Southeast Asia

*Especially with a country like ours that is growing very fast, you bring it down to the economic level. When it is presented in that way, the governor really feels “this is close to my heart,” “it affects my state.” Whatever makes that state prosper is the key, more than always talking about “oooh, healthy children,” you need to add a price tag*.

NGO Broker 028, West Africa

#### Timing

Many brokers planned analyses to coincide with national nutrition policy reviews or program design and attributed this to successful results uptake. Similarly, delayed analyses or a lack of alignment with a specific policy stage were said to have limited the influence of the NMT. However, some consumers of NMTs with multisectoral relevance felt it was more important to strategically apply modeling results when and where needed as policy cycles differed between government ministries.

*The timing for the analysis was so important. It came just as the government had completed the evaluation of the existing nutrition policy and wanted to look at the new policy*.

UN Broker 048, Southern Africa

I think it came a little bit too late. They (government) had actually already planned most of their project already.

UN Broker 076, Latin America

*Maybe it's the wrong timing for one ministry and not the other. For example, Health may not be that open, but for Agriculture it may be perfect timing*.

UN Broker 103, Southern Africa

#### Perceived complexity

Stakeholder understanding of NMT methods and results was critical for adoption and use of the modeling recommendations. Many brokers and consumers considered the NMTs complex to understand and explain to others and thought this hampered government validation or use of their results. Some local technical partners did not feel confident presenting tool outputs as they felt they did not understand it sufficiently or would struggle if asked to answer questions. Modeled estimates of program impact, potential coverage, or diet quality were often misunderstood to be actual figures from observational surveys. Misunderstandings were further compounded when materials were translated into secondary languages. Respondents stressed the need to simplify the tools themselves, to the extent possible, and explain methods clearly, multiple times, using nontechnical language. Some analysts coached local partners to present NMT methods, which strengthened their own understanding as well as informing stakeholders.

*It was critical to get him (government director) on board, being in the key political office he is. We needed him to understand (the modeling) and then explain to his bosses so that they would appreciate where the figures came from and what they meant*.

NGO Broker 104, East Africa

*There's a really big issue around understanding… where these costs come from and the fact that the diets aren't actual diets but that the software optimizes them. That makes it difficult to get a cluster of government people to validate the results*.

NGO Consumer 039, East Africa

#### Ownership

Almost all respondents highlighted local ownership, in terms of stakeholders being involved throughout the analysis process and considering it theirs, as important for NMT results uptake, especially when analyses aimed to influence government policy. Brokers often mentioned ownership when describing their NMT analysis processes and what had been achieved, while government consumers tended to refer to ownership when reflecting on what is needed for successful evidence generation activities.

*All of the institutions put in a lot of time and effort, they weren't just passive stakeholders. It was really important as the results were then from those people in the government, it wasn't information that came in from some international group*.

UN Broker 003, Latin America

*There would not have been much point in doing it without the government ownership because the whole process was geared to change their budget allocation processes*.

UN Broker 025, Southern Africa

*People think that they know what countries need and unfortunately most of the time they don't want to listen and countries just say “okay go ahead, fine.” But if you sit with the government and use their platforms and data to do the modeling, you'd find people would own and absorb and accept that (analysis)*.

Government Consumer 099, Southern Africa

Respondents overwhelmingly agreed that stakeholders were more likely to accept, promote, and apply evidence that they felt reflected their contributions but that agreeing on a shared definition of ownership was critical. Some government end users considered technical involvement in the analysis a prerequisite for ownership, while others were satisfied with a coordinating role.

*If we really want to have national ownership you have to train them (government partners) to produce results themselves*.

Government Consumer 094, Southeast Asia

#### Building capacity to apply NMTs

Representatives from almost all case studies that included capacity building to apply NMTs felt this fostered ownership and understanding, which encouraged the uptake of results. This was the case even where partners were trained but did not do the analysis. In select case studies, NMTs had been institutionalized and were being taught at local universities to public health master's students. This was believed to contribute to sustainable capacity among graduates, many of whom worked in government, to both use the tool and to understand and apply results.

*We always put emphasis on local capacity building, it's more sustainable than bringing in consultants and builds knowledge (on the NMT) so we can work closely with those who will have to move forward with the actions*.

Government Consumer 099, Southern Africa

Some respondents felt local analysts should always be trained in NMTs, while others thought the ability to apply evidence to advocacy and decision making was a more pressing capacity need for local nutrition partners. Many brokers and consumers were concerned that infrequent NMT use or high staff turnover would mean the time and resource investment for capacity building would be difficult to justify.

*We would have to invest a lot of time and funds in building up the analytical capacity of country office staff, this would be useful in the short term but as there is a lot of turnover and you probably wouldn't do it (the analysis) more than once a year, is that really useful?*

NGO Consumer 040, Southeast Asia

#### Secondary data

Data sources were commonly linked to notions of ownership. Most analyses used secondary government data from demographic health, household consumption and expenditure, dietary, or micronutrient surveys. Local partners considered these data high quality because methods were validated, and they were representative at different geographical levels and time points. Some government analysts and consumers felt they owned analyses done with their own data and that secondary analyses validated their work. A few government brokers were wary of modeling done with external data.

*When studies are done by an international agency but using our data, it validates what the government is doing, it gives credence to our work*.

Government Consumer 054, Southeast Asia

*The government ownership was very strong because we saw that it was our data and our people doing it, what we call a “home‐grown solution.” This was the reason for the high acceptance*.

Government Consumer 014, East Africa

Some respondents also shared that analyses based on secondary data facilitated NMT use and, ultimately, policy application, as could be done more rapidly and using less resources than analyses that required primary data collection. In some case studies, it would not have been possible to conduct the analysis if secondary data had not been available, as funds for primary data collection were not available.

*After the initial analysis we received many requests from partners, other countries, etc. This is when we started exploring the possibility of doing the analysis with “big data,” removing the time, cost, and capacity needed for comprehensive data collection*.

NGO Broker 032, Southeast Asia

##### ***External factors***. Capacity for evidence translation and advocacy

Most respondents acknowledged that the modeling could not bring about policy change alone, and that results needed to be translated into advocacy messages and strategically applied to inform or influence decision making. Many flagged capacity needs for such activities within government or NGO nutrition teams. In a few cases, advocacy training was included in the NMT process and said to stimulate broad uptake of the modeling by different government sectors. Elsewhere, individuals with strong advocacy skills were credited for embedding the modeling into government decision making.

*There's no point giving people briefs if they don't have the capacity to do something with them. So, we trained government nutrition focal points, civil society, and academia staff (in advocacy)*.

NGO Broker 104, East Africa

*Instead of training in the modeling itself, it's more urgent to provide better capacity for using evidence like the modeling findings*.

Government Consumer 072, Southeast Asia

*We get a lot of requests to help write the (NMT) results into proposals. Local staff struggle to understand what the modeling says and how to use results to make a case. Doing the analysis isn't enough, they don't know how to then use it in policy*.

NGO Technical Analyst 043, Multiple Countries

##### Resources to support analysis

Government consumers as well as local, nongovernmental partners raised time, staff, and resource constraints as barriers to their meaningful participation in the analysis process and use of results. Even when modeling was done externally, local partners were expected to participate in technical meetings, share data, review results, assist dissemination, and translate evidence into advocacy. Sometimes, suitable government staff could not be identified, were not available, or did not have the management support to do these activities. Occasionally, consultants were hired by local NGO or UN offices or seconded to government offices to support the process. Government partners received funds directly to support NMT applications in rare cases only.

*The ideal “home” for something like this (NMT) would be our national nutrition center but they really don't have the time or capacity, there are no staff to train*.

Government Consumer 005, Southeast Asia

*They (analysts) were asking for several things and this was challenging. We had a lot of other work. Compiling information, translating things, this really takes time*.

UN Broker 061, Central Asia

##### Political change

Political change sometimes meant key partners left their positions or appetite for the modeling was lost. Occasionally, stakeholder engagement had to be restarted and previously validated results or commitments on nutrition were not accepted by new leadership. This was mitigated by planning around political cycles, involving a range of partners and targeting multiple policymakers or sectors.

*Every time they change the government, they change all the way down to the technical staff, so you build capacity, you move a policy process and then everyone leaves and there's no institutional memory*.

NGO Broker 031, Southeast Asia

*The new government had different priorities. The level of interest in overweight and obesity shifted. We will have to be patient and wait to raise this again*.

Government Consumer 010, Latin America

## Discussion

NMTs have been used to generate evidence and inform advocacy and decision making at different policy and program cycle stages in diverse LMICs. The findings provide examples of the policy and program influences NMT applications have achieved, but also suggest that there are opportunities to strengthen the engagement process, capacity to use NMTs, and capacity to apply evidence to ensure that results are used effectively.

NMT applications were mostly driven by international organizations as opposed to being demanded by government decision makers. In the case of more recently developed tools, this may reflect a necessary approach to introduce and build acceptance of novel methods or approaches, similar to the experience of introducing HIV and vaccine modeling tools.^44^, ^45^, ^46^ However, this could also be indicative of broader power imbalances between governments and research institutions in LMICs and donors from high‐income countries or international organizations who can influence research or analysis priorities.^47^, ^48^, ^49^, ^50^, ^51^

Many government end users shared a preference for building domestic capacity to use NMTs and local analysts were trained in some cases. More frequently, national partners reviewed model inputs and outputs, and managed stakeholder engagement. Most analyses had an objective of informing government decision making, often targeting more than one sector or policy cycle stage. Positive influences of the modeling were reported for most cases; from putting nutrition issues on the agenda, to informing specific program components, such as target group selection, transfers, and behavior change messages, to contributing to larger policy changes, including fortification and school feeding legislation.

Almost all respondents acknowledged that changes to policy implementation were not the result of evidence generated by NMTs alone. Instead, strategic advocacy based on the modeling, which often took longer and required more resources than the NMT analysis, was key, as well as complementary evidence generation, such as program costing. Furthermore, respondents credited processes that built understanding and ownership of the modeling with its uptake and use by consumers, as well as conscious planning to make analyses and advocacy relevant, targeted, and appropriate for their intended audiences. However, local partners often required greater capacity and resources to support NMT processes and translate evidence into advocacy and decision making, which limited the reach of the modeling.

As defined by Gillespie *et al*., “enabling environments” for nutrition occur when “political and policy processes that build and sustain momentum for the effective implementation of actions that reduce undernutrition” exist.^52^ Such processes require evidence to encourage best practices, but also leadership, advocacy, technical support, and implementation capabilities, platforms, and resources.^17^ Consequently, given the strength of other forces shaping policy processes, including institutional factors, context, and individual and collective policymaker values, beliefs and culture, evidence generated by NMTs is unlikely to be the sole, or even the most influential, catalyst for substantial policy change.^11^, ^12^, ^14^, ^15^ Indeed, most respondents acknowledged external and complementary advocacy, political processes, and evidence that contributed to the reported NMT policy influences, similar to findings from targeted studies of nutrition policy processes, which also identified the ability to achieve cohesion within policy community as an influential factor.^16^, ^53^, ^54^ This echoes broader discussions in health policy literature that recognizes that evidence is only one of multiple elements influencing policy processes and that, depending on the context, its influence may be outweighed by other factors, such as policymaker beliefs and values.^14^, ^51^, ^55^, ^56^ It is also important to acknowledge the role that policymaker values and context may have in shaping the type of evidence that is generated, which evidence is used, and how it is used.^14^, ^51^, ^55^, ^56^

For the most part, uses of the NMT results were concerned with informing policy decisions, helping to change thinking, or positioning of organizations or topics, in addition to program design. Our findings suggest that NMTs are an important part of a broader ecosystem of evidence generation, capacity building, and advocacy that together build enabling environments for nutrition‐benefitting policy.^14^, ^16^, ^52^, ^57^ Appreciating how NMTs can fit within this ecosystem will ensure they are applied with objectives to identify, complement, and contribute to existing advocacy, in response to endogenous demand, rather than being an end in themselves.

We found 10 factors that influenced how NMTs were used to inform policy and program decisions. Unsurprisingly, similar factors have been highlighted in strategic reviews of evidence use in public health policy, namely, local analysis and advocacy capacity, government ownership or collaboration with decision makers, reputation, targeting and institutional resources, and support.^58^, ^59^, ^60^, ^61^ The importance of targeting evidence generation to align with key policy cycle stages is highlighted throughout health policy literature; however, serendipitous opportunities for influence are also acknowledged.^14^, ^15^, ^62^, ^63^, ^64^, ^65^, ^66^ Lin appreciates that being in the “right place, at the right time, in the right company” for policy change can occur through strategic planning or by accident.^65^ Parkhurst echoes comments from some respondents in pointing out that analysts may not consider the “transfer of knowledge to influence policy” as their role or as within their skillset.^61^ Therefore, political actors or NMT consumers, rather than technical analysts, should carry responsibility for adopting and embedding NMT modeling within targeted advocacy activities.^63^, ^65^, ^67^ At the same time, these political actors or consumers should seek and capitalize on opportunities for serendipitous applications of the modeling results to decision making.^63^, ^65^, ^67^ In both instances, consumer end users, such as government or NGO nutrition focal points, need to understand the policy cycle and be capable of applying evidence to inform and advocate for policy.^53^, ^68^, ^69^

Similar to some respondents in this study, Michaud‐Létourneau *et al*. suggest that building capacity for evidence use may be more pertinent than capacity for evidence generation.^68^ Oliver and Cairney, however, explain that the complexity of the tasks associated with applying evidence to influence policy may extend beyond capacity that can be easily built; the emotional, practical, and cognitive efforts required to advocate for policy change can be substantial.^58^ It may be necessary to recognize the diverse skillsets or technical profiles needed for a successful NMT application and ensure there are resources to involve advocacy, communications, and policy experts, as well as analysts. It is also important to mention that focusing on the capacity needs of individuals risks overlooking the broader institutional structures required to generate and connect evidence to policy.^61^ As such, efforts to build or seek capacity for evidence use and advocacy should consider the rules, resources, and principles through which institutions, such as government and NGO partners, operate.^61^

The perceived complexity of the NMT methods, the tools themselves, or their outputs limited results uptake in some settings. Roberton *et al*. discuss the trade‐offs between the accuracy, and hence perceived complexity, of modeling methods and the usability of the LiST health modeling tool and emphasize the importance of analyst confidence in using health modeling tools and their outputs.^10^ In addition to training and support to use the tools, as discussed further below, the usability of NMT software was raised by some respondents in this study. Anecdotally, tool developers have shared that efforts to make the analysis more accessible to a wider group of less technical users through simplified graphical user interfaces, prepopulated inputs, and time‐saving input and export functions were appreciated. However, as in other studies examining facilitators and barriers to evidence use in policy, respondents put more emphasis on the need to provide high‐quality, simple, and relevant communication of NMT methods and findings to nonanalyst consumers for the modeling results to reach and be used for decision making, rather than a need to simplify or improve the tools themselves.^59^

Meaningful policy impact is often the result of trusting relationships, which can take time and effort to create and maintain, and cannot be rushed or produced during ad hoc workshops.^11^ This has implications for the nature and duration of collaborative relationships and necessary project budgets to maintain these.^70^ Some respondents reported positive experiences in coaching local partners, who were known and trusted by wider stakeholders, to present the modeling themselves, reducing the dependence on international analysts for the interpretation and ongoing use of the modeling.

Competing priorities and insufficient institutional capacity for analysis have been listed as barriers to the local use of other health modeling tools or their results.^10^, ^48^ Practitioners have recommended that necessary resources (time, funds, and capacity) required for the generation, translation, and application of evidence are identified early, and that analyses only proceed if these needs can be met.^53^, ^68^, ^69^, ^71^ This is particularly important given the emphasis respondents in this study placed on involving national institutions and local partners in NMT applications and fostering government ownership. Furthermore, even though definitions of ownership are also ambiguous in the development and public health policy literature, there is general agreement that involving government partners and other stakeholders meaningfully in analysis processes encourages the uptake of evidence for policy decisions.^72^, ^73^, ^74^, ^75^, ^76^ To provide greater clarity and avoid disappointment, Marjanovic *et al*. recommend that stakeholders be invited to jointly define ownership at the beginning of evidence generation processes and that this definition guides the process of working together, as well as sourcing and distributing resources.^77^ It is worth noting that the MAPS tool has employed a “codesign” process consisting of in‐person and virtual workshops in multiple countries.^78^ This is a collaborative process that involves stakeholders and end users throughout the tool's development to ensure that their needs are met and that the final product is useful, valuable, and usable, in addition to promoting ownership.^78^

The existence of a suite of NMTs that can inform policy decisions at different levels of maturity and specificity, for a range of institutions, presents a great resource for LMIC nutrition workforces as well as partners across health, education, social protection, agriculture, and other sectors. In spite of this, and as previously recognized, NMT applications were seldom initiated or led by national governments and were applied in response to government requests in some cases only.^2^, ^10^ The perceptions, values, and interests of nongovernmental stakeholders can influence what is considered trustworthy or quality evidence and which policy or program options are deemed appropriate, feasible, or legitimate.^14^, ^61^ In some of the featured case studies, NMTs were applied among competing ideas or programming options. We can assume that NMT analyses conducted by particular implementing stakeholders or funded by donors with interests in steering government policy discussions in a particular direction may be less impartial than analyses led by governments themselves.^73^, ^79^ Therefore, government ownership and leadership of evidence generation, such as NMT application, should be encouraged to avoid the privileging of external objectives over national interests, in addition to reported benefits for capacity building and evidence uptake.^73^, ^79^

The NMC aims to disseminate information to help end users in LMICs understand tool capabilities and their relevance to specific policy processes.^2^ There is an opportunity to address potential power, resource, and expertise imbalances and to change to demand‐driven, country‐led models in how NMTs are introduced and applied.^80^ This would require financial and technical support for countries to define their own policy questions and be able to consider the tools available to answer them.^2^, ^9^

Unlike most evidence generation discussed in policy literature, many of the NMTs could be applied with or by decision makers in LMICs on an ongoing basis, pending new policy questions or data. This appears to align with the long‐term approach through partnerships with relevant actors to understand the policymaking process recommended by Cairney *et al*., over “one‐off disseminations of state‐of‐the‐art knowledge.”^11^ Similarly, Pelletier *et al*. highlight that the evidence base to support nutrition advocacy should be developed in an “ongoing manner,” rather than only at the beginning of such a process.^70^ While many brokers mentioned the importance of long‐term partnerships with decision makers, one‐off NMT analyses with limited follow‐up were common. In some cases, this was due to the relatively short‐term need for the information some NMTs are designed to generate, while in others, there was a lack of medium‐ or long‐term funding to support the ongoing, collaborative relationships desired by all parties. For NMTs designed to be applied continuously or often, capacity could be institutionalized so that decision makers are supported when needs arise. Tool institutionalization was rare in the case studies, for reasons noted above, yet there were promising examples, such as where NMTs were taught at local universities, leading to demand‐driven, locally managed analyses and a shift in roles for international organizations from “the generation of knowledge to agents of action.”^81^ In some other cases, tool applications were hosted or supported by local government research bodies or international organizations with an in‐country presence. Such organizations were able to apply NMT results to advocacy and decision making continuously. Investigation is needed regarding which tools or policy processes require long‐term, in‐country capacity or hosting, and, for those selected, what resources and circumstances are needed for successful institutionalization and the long‐term sustainability of this.

An alternative would be to make tools as accessible as possible remotely and encourage independent use. However, in their current state, few NMTs can be independently applied without intensive training. While new, preloaded, online tools allow instant access to analysis outputs, they are only designed to answer single analysis questions and are limited in their ability to consider custom data or variations to policy questions.^22^ The ability for self‐teaching via web‐based resources was not mentioned in the analysis, yet is available, to some extent, for a few of the featured tools. Tool developers could support users to run their own analyses by developing self‐guided training materials, videos, or webinars.^10^ Similarly, tools able to be applied without first needing resources for trainings or for hiring consultants to do the analysis or primary data collection would be more accessible to independent users. The feasibility and suitability of independent access and online support for the use of NMTs requires discussion, as does the support needed for end users to identify the appropriate tool(s) for a given policy question.

To our knowledge, this qualitative study is the first to document multiple NMT applications and factors determining their influence on policy or program decision making. As such, the findings provide considerations for planning and conducting NMT analyses, as well as tool development. However, these findings should be considered in light of some limitations. Only case studies that NMT developers knew about were eligible for selection, meaning independent applications of tools available in the public domain may be underrepresented. While quota sampling was used to ensure different end‐user groups were represented, respondents were identified via snowball sampling, which may have limited the range and experience of stakeholders interviewed. There were more case studies from Africa (19) than Asia (7) or Latin America (4), and for three tools, only one case study was identified, so our results may not adequately reflect the experiences of all tools or regions. Furthermore, there was an uneven geographical distribution of end‐user types across regions, based on the participants identified during the snowball sampling. This could mean that responses are somewhat skewed toward areas of higher participation per category, such as Southern Africa and Southeast Asia for consumers and Latin America and Southern Africa for brokers.

Only one researcher (F.K.) conducted the interviews and analysis for this study. While she conducted the interviews with a structured approach, it is possible that some insights were missed or overstated. Moreover, as the researcher used RTA, the development of themes was influenced by her background and knowledge of the subject and may have differed if the analysis were done by others.^42^ Lastly, individual tools and case studies were not identified in the analysis to avoid comparison of tool methods or implementing organizations and to preserve anonymity, as the focus of the paper was on the process of applying NMTs rather than the tools themselves. This limited the ability to describe key tool elements in detail and any relevance they had for policy influence.

We found that NMTs have had wide‐ranging influences across policy and program cycles and contexts, contributing to an enabling environment for nutrition, but that the extent of these is highly dependent on the tool application process, accompanying advocacy, and the expertise, capabilities, and resources of local teams. Understanding how NMTs have been applied and what was achieved as a result could assist potential end users to appreciate the policy questions these tools can address, as well as make a case for allocating resources for such analyses by demonstrating their policy value. We also found that potential remains for even stronger policy influence. The factors explored in this study can inform how NMTs are applied in the future, including planning the analysis process, materials to support NMT applications, modifications to existing tools or new tool development, and resourcing for local collaborator participation and evidence translation in all of these activities.

## Author contributions

All authors conceptualized the study. F.K. conducted the interviews, carried out the analyses, interpreted the results, and wrote the manuscript. All other authors contributed to and approved the final manuscript.

## Competing interests

The authors declare no competing interests.

### Peer review

The peer review history for this article is available at: https://publons.com/publon/10.1111/nyas.14778

## Supporting information


**Table S1**. Nutrition modeling tools examined as part of this study
**Table S2**. Interview guide
