# Supplementary material for: Nutrition modeling tools: a qualitative study of influence on policy decision making and determining factors
Source: Ann N Y Acad Sci. 2022 Apr 20;1513(1):170–91. doi: 10.1111/nyas.14778 (PMC9546113; doi:10.1111/nyas.14778)
Supplement: Supplementary file 1 — Table S1. Nutrition modeling tools examined as part of this study Table S2. Interview guide [file NYAS-1513-170-s001.docx]

Supplementary Table 1:Nutrition Modelling Tools examined as part of this study

| **Tool** | **Parent Organisation/s** | **Brief Description** | **Tool Format** |
| --- | --- | --- | --- |
| **Cost of the Double Burden** CODB | Economic Commission for Latin America (ECLAC) | Analyses the potential social and economic cost of the double burden of under- and over nutrition and estimates how much could be saved by addressing this. | Templates populated in country and completed by ECLAC team |
| **Cost of Hunger** COHA | African Union (AU) and Economic Commission for Latin America (ECLAC) | Analyses the potential social and economic cost of under nutrition and estimates how much could be saved by addressing this. | Templates populated in country and completed by AU and ECLAC teams |
| **Cost of Not Breastfeeding** CoNBF | Alive and Thrive | Estimates the human and economic costs of not breastfeeding for 30 countries. Provides estimates of preventable child morbidity and maternal and child mortality to inadequate breastfeeding as well as cost implications for households, the health system and economic productivity due to mortality and cognitive losses. | Online, pre-loaded dashboard |
| **Cost of the Diet** CotD | Save the Children UK (SCUK) | Models the lowest-cost, nutritionally adequate diet for model households and individual target groups, based on the nutrient requirements of each household member and locally available foods and their costs. | Software Programme |
| **Fill the Nutrient Gap** FNG | World Food Programme (WFP) | Extends on SCUK’s CotD methods by estimating the extent to which households would be able to afford nutritious diets and modelling the potential impact of different nutrition-sensitive and specific interventions to improve access to nutritious diets. | CotD software and analysis framework, completed by WFP team |
| **Intake Modelling, Assessment and Planning Program**  IMAPP | University of California Davis | Supports the 2006 WHO / FAO Guidelines on Food Fortification using current consumption of potential fortification ‘vehicles’: what amount of nutrient to add for a targeted prevalence of inadequacy? | Software Programme |
| **The Lives Saved Tool – Nutrition** LiST | Johns Hopkins University | Models how changes in coverage of efficacious interventions affect mortality, stunting, wasting, breastfeeding practices, birth outcomes and maternal anaemia. | Software Programme |
| **Micronutrient Action Policy Support tool** MAPS | University of Nottingham and London School of Hygiene & Tropical Medicine (LSHTM) | Enables access to subnational, spatially-disaggregated data on the micronutrient status of populations including biomarker data and dietary supply data for nations in sub-Saharan Africa. Options to improve dietary micronutrient supply through consumption or composition changes can be explored, included a limited set of costed interventions, while the tool will also present projections of dietary micronutrient supplies to 2050. | Open-source tool (under development) |
| **Micronutrient Intervention Modelling Project** MINIMOD | University of California Davis | Makes use of primary or secondary data on dietary intake or food expenditures. Identifies inadequate intake of MNs and cost-effective solutions to specific MN-related problems. Includes a cost tool to compute total program costs and the cost per individual reached or effectively covered, and an economic optimization tool that identifies cost-effective combinations of programs over time/space, given budget constraints. | Software programs |
| OPTIFOOD | London School of Hygiene & Tropical Medicine (LSHTM) | Identifies the extent to which local food environments and dietary patterns can provide nutritious diets. Identifies problem nutrients and strategies to address gaps using food-based recommendations. | Software Programme |
| PROFILES | FHI 360 and USAID | Raises awareness among policymakers of the consequences if malnutrition does not improve over a defined time period and the benefits of improved nutrition over the same time period, including lives saved, disabilities averted, human capital gains and economic productivity gains. . | Excel-based templates |
| **Strengthening Economic Evaluation for Multisectoral Strategies for Nutrition** SEEMS | University of Washington | Generates robust and reliable data on costs and benefits of multisectoral nutrition programs, making analyses on the cost-effectiveness of programs and the return on investment possible. This enables decision makers to make informed choices regarding new investments, scale-up opportunities, prioritization of funds and more. | Under development |

Adapted from Bergeron 2018 (2)

Supplementary Table 2: Interview Guide

| **Area** | **Interview Questions** |
| --- | --- |
| A. Details of case study (tool application) | 1. Can you please tell me about the role that you had in the _______ (country) tool application?  **Probes:** What was your position/organisation at the time?  What role did you play in setting up/conducting/overseeing/disseminating results from or acting upon the analysis?  2. What led to the decision to use the ____(tool) in _______ (country)?  **Probes:** What was the question that the analysis was trying to answer?  Was there any particular reason why the ____(tool) was selected over another type of analysis or tool?  3. What were the objectives of the ____(tool) application?  **Probes:** Were there any specific analysis questions?  Why was the tool used when it was?  Did the ____(tool) application fit in to any policy or programme planning cycles? |
| B. Stakeholder identification and engagement | 4. Who was involved in the process of applying the ____(tool) in _______ (country)?  **Probes:** Who led the process in country?  Who funded the analysis? Why?  Who actually carried out the analysis?  Was anyone else involved?  5. Who were the important stakeholders in this process and what was done to identify and engage with these stakeholders?  **Probes:** Who were the Government stakeholders? Which sectors were represented? What level of government was targeted (directors, ministers, president)?  Who were the target audience for results?  UN organisations?  NGOs?  Civil Society?  Other? |
| C. Process for planning and carrying out analysis | 6. Can you tell me how the analysis itself was planned?  **Probes:** What decisions were made and who made these decisions?  How were the priorities for the analysis set?  What was involved in getting support for the process?  Was any training provided?  7. How was data identified or collected for the analysis (if relevant)?  **Probes:** What was needed to do this?  Were quality data available?    8. How was the data analysis carried out and who was involved?  **Probes:** Who was responsible?  Did anyone else contribute to the analysis?  Was a technical working group formed?  Was there any process of stakeholder validation of data/results as they emerged?  9. What was needed (capacity, funding, training, software etc.) to do this analysis and where did the resources and capacity come from? Were there any differences between what was needed and what was provided?  **Probe for:** Human capacity, resources, time, etc. |
| D. Modelling Results | 10. Can you tell me about the main results or findings from this analysis?  **Probes:** Were any of these findings more important or interesting than others? Why?  Were these results expected or were any surprising to you/others? Why?  To what extent were these findings *novel*? Why/why not? |
| E. Process for disseminating and applying findings | 11. What was the process used to share the modelling amongst stakeholders?  **Probes:** Were there any particular objectives of this process?  What methods were used (meetings, workshops, creation of policy briefs etc.)?  12. Who was involved in this dissemination process and what was their role?  **Probes:** Who were the champions for the modelling?  Who were the leading organisation/s?  Were these the same stakeholders involved earlier, during the analysis?  13. Were there any particular actions that were carried out/planned to apply these results to advocate for action?  **Probes:** What methods were used (meetings, workshops, creation of policy briefs etc.)? |
| F. Policy Influence | 14. What was the reaction to the modelling or calls for action from the stakeholder community?  **Probes:** What was the reaction from Government at the technical level (by sector)?  Government at the decision-making level (by sector)?  Local non-government partners?  International organisations?  Others?  15. To what extent were the objectives (from question 3) of this analysis met?  **Probes:** Can you tell me about anything that has changed or is in the process of changing as a  result?  16. Can you tell me any other examples of how the modelling has been used by your organisation and/or decision-makers at government or other organisations?  **Probes:** Can you tell me about anything that has changed or is in the process of changing as a  result?  Policies, programme decisions, investment?  Probe by different government sectors.  17. What would a successful implementation and influence of the ____(tool) look like in your opinion? To what extent did this occur in your setting? Why/why not?  **Probes:** Knowledge and opinions, partnerships, advocacy messages, changes to policy or programming processes, implications for other sectors. |
| G. Influencing Factors or Enabling Environment | 18. To what extent was the *scientific approach* used for the ____(tool) analysis accepted in (country)?  **Probes:** Did this differ by stakeholders/stakeholder groups?  Did this approach assist in engaging stakeholders or the acceptance of results?  What was done to help stakeholders understand this approach?  19. To what extent was the *process used to carry out the* ____(tool) *analysis and work* with stakeholders accepted in _____ (country)?  **Probes:**  Which elements of the process?  Did this help or hinder how the modelling was accepted, understood and used?  What could have been done better?  20. How satisfied do you think that people were with the dissemination process?  **Probes:** What about the results, advocacy messages and calls for actions?  How satisfied were you/your organisation?  How satisfied were other stakeholders?  What could be done better?  21. What other factors determined how successful the use of the ____(tool) was in ­­­­­_________(country) in terms of influence on policy or programme decisions? What impact did these factors have?  **Probes:** Resources available/allocated, leadership and staff capacity, data access, stakeholder engagement and participation, communication, time available/taken, processes used, complexity of tool/results and understanding of these, timing of tool allocation and political climate for nutrition. |
| H. Future use | 23. If the ____(tool) was to be used again here or in another country, what would you change?  **Probes:** Would you use this tool again?  Who should use this tool?  How should it be used?  24. What do you see as the ‘future’ of the ____(tool) ?  **Probes:** Institutional positioning?  Regional/local capacity building?  Government ownership?  Follow-up analysis in the country? |
